# What Makes a Good Palliative Care Physician? A Qualitative Study about the Patient’s Expectations and Needs when Being Admitted to a Palliative Care Unit

**DOI:** 10.1371/journal.pone.0158830

**Published:** 2016-07-07

**Authors:** Eva K Masel, Anna Kitta, Patrick Huber, Tamara Rumpold, Matthias Unseld, Sophie Schur, Edit Porpaczy, Herbert H Watzke

**Affiliations:** 1 Department of Internal Medicine I, Division of Palliative Care, Medical University of Vienna, Vienna, Austria; 2 Department of Internal Medicine I, Division of Hematology and Hemostaseology, Medical University of Vienna, Vienna, Austria; Tokai University, JAPAN

## Abstract

**Objective:**

The aims of the study were to examine a) patients’ knowledge of palliative care, b) patients’ expectations and needs when being admitted to a palliative care unit, and c) patient’s concept of a good palliative care physician.

**Methods:**

The study was based on a qualitative methodology, comprising 32 semistructured interviews with advanced cancer patients admitted to the palliative care unit of the Medical University of Vienna. Interviews were conducted with 20 patients during the first three days after admission to the unit and after one week, recorded digitally, and transcribed verbatim. Data were analyzed using NVivo 10 software, based on thematic analysis enhanced with grounded theory techniques.

**Results:**

The results revealed four themes: (1) information about palliative care, (2) supportive care needs, (3) being treated in a palliative care unit, and (4) qualities required of palliative care physicians. The data showed that patients lack information about palliative care, that help in social concerns plays a central role in palliative care, and attentiveness as well as symptom management are important to patients. Patients desire a personal patient-physician relationship. The qualities of a good palliative care physician were honesty, the ability to listen, taking time, being experienced in their field, speaking the patient’s language, being human, and being gentle. Patients experienced relief when being treated in a palliative care unit, perceived their care as an interdisciplinary activity, and felt that their burdensome symptoms were being attended to with emotional care. Negative perceptions included the overtly intense treatment.

**Conclusions:**

The results of the present study offer an insight into what patients expect from palliative care teams. Being aware of patient’s needs will enable medical teams to improve professional and individualized care.

## Introduction

Palliative care involves a multidisciplinary and individual approach, consisting of professional care in the form of optimum symptom control, psychosocial and spiritual care and support in the organization of everyday life. Palliative care is known to improve quality of life, but the patient’s referral to a palliative care unit is still hindered by several barriers, including lack of ward capacity and the unwillingness of physicians to refer their patients into this type of care [1–6]. In Austria, palliative care is at the moment insufficiently represented, as only a low percentage of patients can have access to a palliative care unit. Besides structural, administrative, and economic reasons, there are several other factors contributing to the underrepresentation of palliative care in health politics. These include both patients and physicians‘ uncertainties about its exact purpose, physician‘s uncertainty concerning the prognosis of the patients and emotional barriers of some physicians to initiate end-of-life-conversations due to the fear of undermining the patient's hopes of recovery [7]. From our experience as palliative care physicians, patient’s referral to our unit occurs much too late, as we see that patients on our waiting list often die before having the chance to be admitted to the palliative care unit. Recent studies addressing public awareness of palliative care show that members of the public identify palliative care with caring for people who are dying and ensuring their comfort in the terminal phase of their lives. Expectations of carers concerning palliative care have also been investigated, showing that advance care planning and positive communication may help to optimize the care of severely ill patients, and additionally support their relatives [8–10]. However, we know far too little about the patient’s view of palliative care. Just a handful of studies have been published in this field, and these were focused on the preferred place of death, communication, and the influence of cultural backgrounds [3, 11–17]. Patients’ preferences in palliative care revealed four key aspects: living a meaningful life, responsive health care personnel and care environment, and responsiveness in the organization of palliative care [18]. Since a large percentage of cancer patients may be expected to have palliative care needs, it is imperative to know more about what those patients expect from their ward and the attending medical staff [12, 13, 19]. In Austria, specialized hospice and palliative care have not yet been fully integrated into the traditional health care system. Austrian palliative care units are small, hospital-based units comprising 8 to 14 beds. Focused primarily on cancer patients, their principal task is to support patients with advanced cancer and their caregivers until the patients can be discharged from the hospital. Nevertheless, according to the European Association for Palliative Care (EAPC), mortality rates in Austrian hospitals are as high as 25%. A distinct feature of hospices—unlike palliative care units—in Austria is that they are not regularly funded by the state. Their purpose is to accompany the patients in the final phase of life until their death. Given the small number of hospice beds, a mere 10% of the patients die in hospices [20]. The present study aimed to investigate how much patients know about their upcoming palliative care, their expectations and needs when being admitted to a palliative care unit, how patients define a good palliative care physician and how their attitude changes during admission. The data were analyzed and the results discussed with a view to preparing patients as well as caregivers prior to admission to a palliative care unit.

## Materials and Methods

This study employed a qualitative design using semistructured interviews. The interviews were conducted using a number of predetermined open-ended questions (Table 1). The method permitted the participants to respond in their own words and set their own emphases while providing a frame of orientation and not omitting important issues. At the end of each interview, the interviewer ensured that all topics had been addressed during the interview [21].

**Table 1:** Interview Guide for the first and second Interview.

First interview
**How old are you and where do you come from?**
**You are being admitted to the palliative care unit for the first time. How have you been since you arrived?**
**What do you associate with a palliative care unit?**
**Can you remember the moment you were asked about being admitted to a palliative care unit? When was it and how did you feel?**
**Had you heard anything about palliative care before that? Did you have any idea what it is?**
**What did your treating physician tell you in this regard?**
**What do you consider the most important aspect of this unit?**
**What do you expect from the physicians here?**
**Is there anything special you would wish of the physicians here?**
**How would you define a good physician?**
**Is there something else you would like to communicate about the subject?**
**Second interview**
**It’s now a week since you were admitted to the palliative care unit. How are you doing since then?**
**I asked you this question last week. Now I am interested in knowing whether your point of view has changed, so I’ll ask you the question again: What is your concept of palliative care?**
**What, in your view, is the most important aspect of this unit?**
**What was the most pleasant surprise you encountered during your treatment at this unit?**
**What was the most unpleasant surprise you experienced during your treatment?**
**When you look back at the moment you were first asked about being admitted to a palliative care unit and what you associated with palliative care at the time: do the expectations and ideas you had at the time match your experiences during the last week?**
**What do you expect of the physicians here?**
**Were there situations during the last week when you were dissatisfied with the medical treatment or had needs that were not met?**
**When you look back at other hospital stays and compare them to the last week at the palliative care unit: did the treatment differ in any way?**
**How would you define a good physician?**
**Is there something else you would like to communicate about the subject?**

### Participants and Data Collection

The investigation was performed at the palliative care unit of the Medical University of Vienna, which is a hospital-based facility consisting of 12 beds mainly attending to cancer patients. Inclusion criteria were age over 18 years, no severe reasons for non-participation (cognitive deficit, delirium, severe mental illness, severe septicemia), no language problems and the ability to give written informed consent. The Karnofsky performance status scale (KPS) was used to rate patients' functional status on the day of admission by the palliative care physician in charge as part of the medical history. Participants gave their written consent to their inclusion in the study and digital recording of their interviews. Patients who did not meet the inclusion criteria, were not able to communicate properly or did not give their consent were excluded from the study. Interviews were conducted between May 2014 and May 2015. Caregivers were allowed to be present during the interviews. The duration of the interview ranged from 6 to 52 minutes (mean 25 minutes). To minimize bias, all patients were interviewed by a female medical student (A.K., with a bachelor’s degree in social and cultural anthropology) who was not a team member. The patients were interviewed twice. The purpose of the first interview was to examine their knowledge of palliative care, their expectations and perceived needs on admission to the palliative care unit and their concept of good palliative care physicians. The first interview was held within the first three days after admission and the second interview after one week. The purpose of the second interview was to record the patients’ experience after being admitted to the palliative care unit, and whether their perception of palliative care had changed.

### Data Analysis

The interviews were conducted in German and transcribed by a professional transcriber. Two female researchers with experience in qualitative research analyzed the interview transcriptions by open coding (A.K. and E.K.M.). Neither of them had established a relationship with the study participants prior to the study. The German statements were transcribed by a professional transcriptionist and translated into English by a professional translator. As a translation always involves a certain loss of originality, particular attention was given to conscientious and careful translation. Thematic analysis was applied using a combination of inductive and theoretically driven techniques enhanced with grounded theory techniques [22, 23]. Grounded theory techniques were used involving open-ended questions, iterative coding, line-by-line coding, constant comparison, and memo-writing throughout the analysis process. We combined those techniques because we aimed to reach a deeper understanding of the answers to our research questions. Emergent themes were developed following a series of coding steps: first, open coding was used and initial codes were generated. Next, initial codes were grouped into according to their similarity. Then, these categories were organized into themes. The coding process started after transcription of the first interviews and was continued through the entire coding/analysis process. To ensure quality, special emphasis was placed on the meaning the patients assigned to their experiences and to factors that might have influenced their perspectives and interactions as pointed out in the Interview Guide [24]. The interviews had an informal character while open questions helped to focus on topics that seemed most important to the patients. When a patient had difficulties in understanding the questions or raised sensitive topics, the interview and its language were modified to the individual situation. The transcripts of the interviews were not returned to participants for comments or correction. The software program NVivo 10 was used for analyzing the data. To ensure the reliability of the coding process, two researchers (A.K. and E.K.M.) independently generated a list of codes after reading the interviews. The results were then compared. There was high agreement between the encoders; differences were resolved by verbal discussion with three other researchers (M.U., P.H. and S.S.). The themes were identified by group discussion until consensus about the interpretation of the themes was achieved. The investigators compared their findings, discussed categories, and agreed upon the coding. This study was approved by the Medical Ethics Committee of the Medical University of Vienna (Approval no. 1355/2013).

## Results

### Patient Characteristics

**Fig 1:**
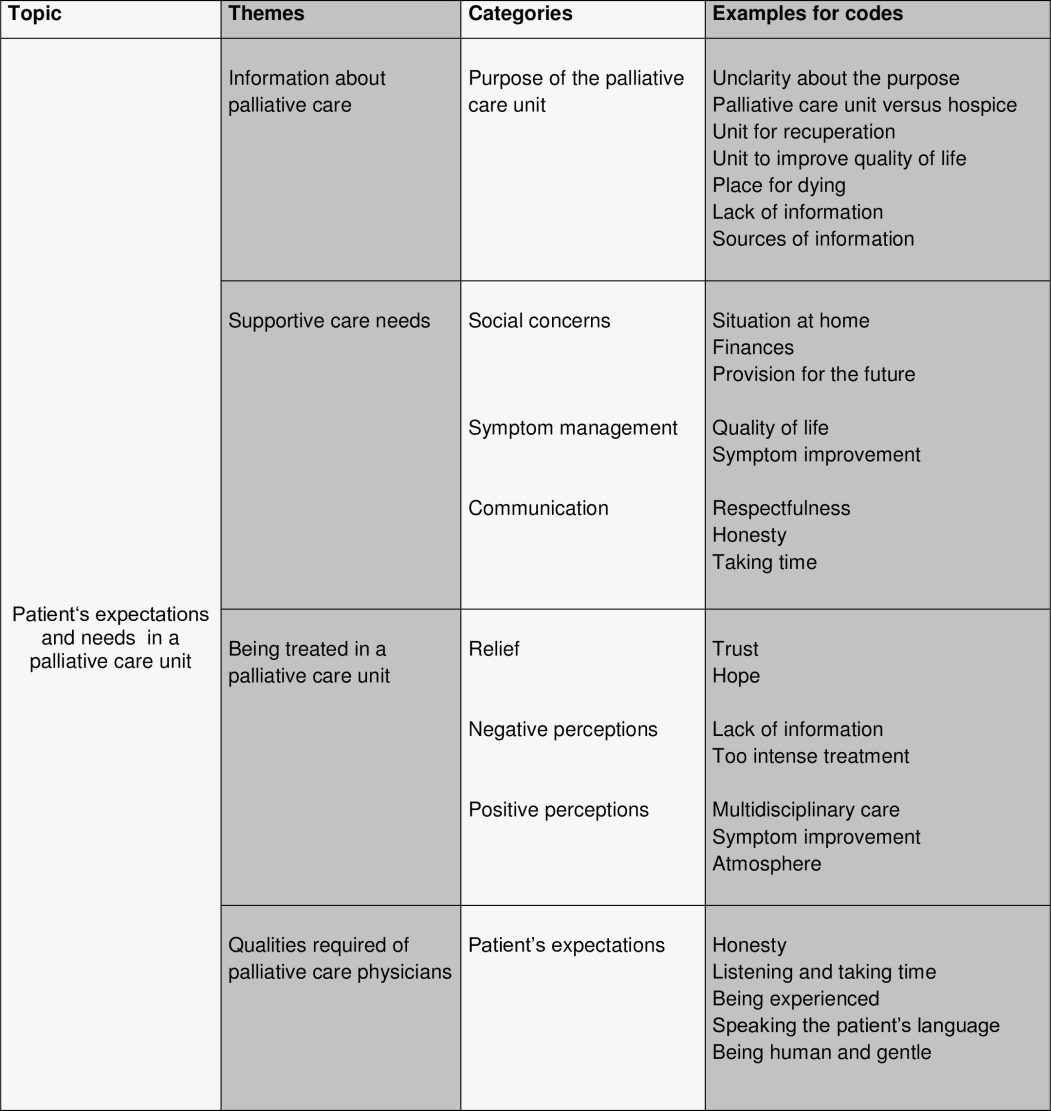
Themes, categories and codes generated from patient interviews. Fig 1 depicts the themes, categories and examples for codes identified from patient interviews. Codes relate to the patients‘ knowledge of palliative care, their expectations and perceived needs when being admitted to a palliative care unit, and their concept of a good palliative care physician.

Thirty-two interviews were conducted with 20 patients suffering from advanced cancer, selected from a larger cohort of 45 patients. Twenty-five patients had to be excluded for the following reasons: a) Twenty-two did not meet the inclusion criteria (nine patients had an excessively poor performance status, four patients were suffering from a severe cognitive deficit due to brain metastases, three patients suffered from delirium, three patients had severe septicemia, two patients had language problems, and one patient had a cognitive impairment due to hypercalcemia, b) three patients did not consent to participate in the study. Demographic characteristics of the twenty included patients were as follows: 65% women, 35% men; median age 65 years (range, 42–85 years); median Karnofsky performance status scale (KPS) on admission 60% (range, 40–80 percent). The reasons for hospitalization were deterioration of performance status because of multiple symptoms (45%), uncontrolled cancer-related pain (30%), dyspnea (15%), and psychosocial factors (10%). Twelve patients completed both interviews (P1, P3, P5, P6, P7, P9, P10, P13, P14, P16, P17, P19), four died prior to the second interview (P2, P4, P18, P20), and further four could not participate in the second interview because their performance status had severely deteriorated since the first interview, but still consented to usage of the first interview (P8, P11, P12, P15). The caregivers stayed with the patients during two interviews (P6, P13). All patients consented to the use of the interviews for the study. When theoretical saturation was achieved and the analysis of the most recent interviews did not generate new themes or codes, no further participants were enrolled. Sociodemographic characteristics and general cohort statistics are shown in Tables 2 and 3. The themes, categories and examples for codes generated from patient interviews are shown in Fig 1. The original German quotations are mentioned in Supporting Information (S1 Appendix).

**Table 2:** Profile of the Study Participants.

Participants	Age	Sex	Oncological	Performance
	(years)		disease	Status (%)
P1	58	f	Breast cancer	50
P2	73	m	Lung cancer	60
P3	64	m	Brain tumor	40
P4	53	f	Breast cancer	70
P5	42	f	Small Intestine cancer	70
P6	74	m	Lung cancer	60
P7	66	f	Lung cancer	70
P8	68	f	Pancreatic cancer	50
P9	49	f	Breast cancer	70
P10	75	m	Lung cancer	80
P11	70	f	Rectal cancer	80
P12	69	f	Breast cancer	60
P13	51	f	Sarcoma	70
P14	72	m	Bladder cancer	50
P15	85	m	Liver cancer	70
P16	81	f	Pancreatic cancer	60
P17	69	f	Lung cancer	50
P18	51	m	Skin cancer	50
P19	79	f	Pancreatic cancer	50
P20	59	f	Breast cancer	60

**Table 3:** Baseline Characteristics of Patients.

Baseline Characteristics of Patients, n = 20			
		n	%
Sex	Female	13/20	65
	Male	7/20	35
**Age**		median	range
Years		65	(42–85)
**Performance status**		60	(40–80)
**Histology**		n	%
Breast cancer		5/20	25
Lung cancer		5/20	25
Pancreatic cancer		3/20	15
Bladder cancer		1/20	5
Brain tumor		1/20	5
Small Intestine cancer		1/20	5
Liver cancer		1/20	5
Rectal cancer		1/20	5
Sarcoma		1/20	5
Skin cancer		1/20	5

## Qualitative Findings

The analysis of the interview transcripts revealed four themes: information about palliative care (theme 1), supportive care needs (theme 2), being treated in a palliative care unit (theme 3), and qualities required of palliative care physicians (theme 4). The themes jointly address the patients’ expectations and perceived needs when being admitted to a palliative care unit and their concept of a good palliative care physician.

The first interview is marked Pxa (patient number x/interview a) and the second interview Pxb (patient number x/interview b). The results are presented narratively, exploring each theme and its key features using patient quotations.

### Theme 1: Information about Palliative Care

#### There is unclarity about the exact purpose of the palliative care unit

When their admission to a palliative care unit was suggested, most patients were aware of hospices. They were unable to exactly distinguish palliative care units from hospices. *“One reads or hears about hospices in the newspaper or on television*. *I thought a palliative care unit was the same thing” (P01a*, *female*, *aged 58 years*, *suffering from breast cancer)*. Three participants had been told by their treating physicians that care in a palliative care unit would be more intimate, patient-centered and intensive, that the staff would take more time, and their quality of life would improve, as one participant explained. *“I had heard that one deals differently with the situation of a diseased person and is more intensively focused on it” (P03a*, *male*, *aged 64 years*, *suffering from brain tumor)*. In general, many patients (twelve) had little prior information about the palliative care unit. Eight patients had the impression that their stay on the palliative care unit would primarily serve to “coddle them up”as stated by their treating physicians prior to their admission. The primary perception of the palliative care unit on admission varied among patients. Seven interviewees perceived the palliative care unit as a hospice or a place where they were probably going to die, as one participant put it. *“Conceptually I never really got it*. *In rural areas*, *where I live*, *‘palliative’ is something that gives you the idea of having reached the end of it all*. *But apparently people want to help you here” (P04a*, *female*, *aged 53 years*, *suffering from breast cancer)*. Ten patients were uncertain as to whether the purpose was to discharge them or whether their admission to the palliative care unit meant that they were going to die soon. *“Palliative*, *in colloquial language means ‘Palliative*, *the last little chamber of your life*!*’ But that’s probably not true” (P19a*, *female*, *aged 79 years*, *suffering from pancreatic cancer)*. Two patients felt their admission was wrong in terms of incorrect estimation of the severity of their disease or due to a shortage of beds in other units. Eight patients thought it was a unit for recuperation and symptomatic relief, whereas four patients did not know what a palliative care unit actually is. *“I had absolutely no idea*. *Even now I have no idea what I’m doing here and what’s going to happen” (P02a*, *male*, *aged 73 years*, *suffering from lung cancer)*. *“I haven’t been told what it is*. *One has to ask*! *(laughs)” (P09a*, *female*, *aged 49 years*, *suffering from breast cancer)*. Some patients were informed about palliative care because they had heard about it on TV or from their friends and family, had read brochures on it or had consulted the Internet.

### Theme 2: Supportive Care Needs

#### Patients require help in social concerns, symptom management as well as respectful communication

Thirteen patients expressed a great need for assistance in social concerns in the form of regulating their situation at home, their finances and their provision for the future. *“Social pressures are given too little attention because those are factors that impose a terrible burden on human beings”* ***(****P03b*, *male*, *aged 64 years*, *suffering from brain tumor)*. Maintaining an active social life and improving quality of life was expressed as an achievable goal. *“I’ve heard this is actually a unit for the dying*. *Some survive it and return home and experience better quality of life*. *That would be very nice*. *I hope I’m able to do that too” (P07b*, *female*, *aged 66 years*, *suffering from lung cancer)*. Attentive communication and qualities such as listening and respectful treatment were very important to the patients: *“(…) Taking me seriously*, *yes*. *And listening*, *yes*. *I always wanted that*. *Sometimes I had the impression that doctors don’t listen to me at all”* ***(****P05a*, *female*, *aged 42 years*, *suffering from cancer of the small intestine)*. It seemed that the patients had experienced different types of communication in the course of their disease and wanted their situation to be dealt with in a sensitive way. *“Taking a bit of time*, *listening*, *listening and showing some empathy*, *but not saying something like*, *‘Well*, *I don’t think you’re going to be around very long anyway’”* ***(****P19b*, *female*, *aged 79 years*, *suffering from pancreatic cancer)*. Honesty and information about the disease was a high priority for participants and, in addition to professional care, taking time was mentioned as particularly valuable. “*These special characteristics are competence—which I consider very important—and communicating what’s happening*. *And what the prognosis of the disease is—since I’m asking*, *I should know*. *Honesty and continued efforts to try the best options in my case”* ***(****P20a*, *female*, *aged 59 years*, *suffering from breast and ovarian cancer)*. A further need was the alleviation of distressing symptoms. Seven patients hoped their symptoms would improve during their stay at the palliative care unit. “*I associate palliative medicine with the very last phase of life and the fact that someday—when I’ve reached the goal—I’ll be able to live a life without pain” (P10a*, *male*, *aged 75 years*, *suffering from lung cancer)*.

### Theme 3: Being Treated in a Palliative Care Unit

#### After one week, the patients experienced relief while being treated in a palliative care unit

The patients were interviewed after one week and asked whether their perception of a palliative care unit had changed after their admission. At this point in time the patients perceived their admission to a palliative care unit as something positive. Rather than giving up hope for improvement, they realized palliative care is a possibility to get the most appropriate care. *“Now I know that a palliative care unit is a place where they get you into a fit condition to go home*. *Yes*, *and one can return here anytime if one gets worse” (P01b*, *female*, *aged 58 years*, *suffering from breast cancer)*. The patients expressed trust towards the palliative care team and five patients reported that the stay at the palliative care unit had helped them to regain hope through improvement of burdensome symptoms, by which they had been both physically and mentally impaired in their daily lives. *“Getting back some strength and gaining hope—that has been achieved here*.*”(P19b*, *female*, *aged 79 years*, *suffering from pancreatic cancer)*. The patients had had the opportunity to talk to different members of the palliative care team and realized that their being discharged was one of the foremost aims of the palliative care unit. Five patients expressed the notion that the staff at the palliative care unit would be more accesible to them than those at other wards. *“I think one takes more care of people here*. *They’re not necessarily focused on your health alone*, *but ensure you get some rest here*, *or at least a break*. *They offer support in situations that are needed*, *for example*, *psychologists*, *dieticians*, *or what is necessary at home*.*” (P03b*, *male*, *aged 64 years*, *suffering from brain tumor)*. The patients’ perception had changed in the way that their association of palliative care with dying was superseded by a more positive attitude towards this type of care. *“I thought it would be worse*. *I thought the people there are very ill*, *but I thought it would be worse and nobody would be able to leave this unit anymore” (P09b*, *female*, *aged 49 years*, *suffering from breast cancer)*. Six patients reported that they had not associated a palliative care unit with this type of treatment before their admission, but would have benefited from the attention through the palliative care team involving different professional groups. *“I had no idea what a unit of this type was and I’m really pleasantly surprised to see how concerned people are here and how they do their work*. *It’s different from other places and that’s probably how it should be*. *I think it’s the right way” (P07b*, *female*, *aged 66 years*, *suffering from lung cancer)*.

#### Negative perceptions: lack of information about palliative care and too intense treatment were criticized

Compared to their initial impression the patients wished they had been better informed about the purpose of their stay in a palliative care unit prior to admission. Prior information, they said would have given them another contact point in addition to their treating oncologists. *“Being told some day—I visit the oncology department so often*, *in fact every three weeks—would’ve been good if they’d told me*, *‘Listen*, *if you start to feel worse*, *there’s a special unit there*,*’ and so on*. *But they never spoke about it*. *They never said a word about it” (P01b*, *female*, *aged 58 years*, *suffering from breast cancer)*. Some patients complained that the treatment was too intense and too many members of the palliative care team were visiting them. *“The psychologist comes*, *then the voluntary workers come*. *Sometimes it’s just a bit too much” (P13b*, *female*, *aged 51 years*, *suffering from sarcoma)*.

#### Positive perceptions: multidisciplinary, attentive care and symptom improvement were perceived as positive

The patients acknowledged that members of the multidisciplinary palliative care team took time to tend to their needs, and that burdensome symptoms were improving. Emotional care, welcoming atmosphere and support were regarded as positive components. *“Not just ‘Yes*, *yes*, *we’ll do it’ and nothing following the statement*. *Rather*, *something is done and it helps*, *yes” (P03b*, *male*, *aged 64 years*, *suffering from brain tumor)*. The easy availability of the attending staff was rated positively. *“When one says*, *‘I want to talk to a doctor*,*’ then there’s someone available the whole week who explains everything to you*. *(…) Simply time*. *They take time and explain everything carefully” (P05b*, *female*, *aged 42 years*, *suffering from cancer of the small intestine)*.

### Theme 4: Qualities Required of Palliative Care Physicians

#### Patient’s expectations of a good palliative care physician

On being asked about their notion of a good palliative care physician, some patients started to narrate their negative experiences about being treated dishonestly by physicians in a former care setting. Honesty was particularly important to the patients. *“Honesty*. *I don’t need to ponder on it*. *That’s it*: *simple honesty” (P18a*, *male*, *aged 51 years*, *suffering from skin cancer)*. The interviewees mentioned the following core aspects of a good physician tending to patients with advanced cancer: honesty, the ability to listen, taking time, being experienced in their field, speaking the patient’s language, being human, and being gentle. As one participant explained at his second interview, patients realized that a palliative care unit does not merely deal with dying, but is a place where attention is given to the patient’s resources and the improvement of the symptoms of severe diseases. *“Good news maybe and yes*, *I think the doctors here are like that*. *They laugh a lot (…)*, *yes*, *doctors should laugh more than they do” (P05b*, *female*, *aged 42 years*, *suffering from cancer of the small intestine)*. Most patients desired their treating physicians to take time and some had experienced a lack of time in clinical practice, as mentioned by two patients. *“Taking time*, *communicating*, *listening and being humane*. *It’s very*, *very important to listen” (P07b*, *female*, *aged 66 years*, *suffering from lung cancer)*. During their previous experiences, some patients had experienced busy personnel and missing resources: *“A little more time for patients*. *A little more time*. *That’s practically non-existent*. *When you go to the outpatient services you’ll see—they’re all overcrowded*. *How in the world are they going to spend time?* *(…) I think that’s monitored by the politicians*. *The working hours are being cut all the time and nothing sensible comes of it*. *I don’t understand politics” (P08a*, *female*, *aged 68 years*, *suffering from pancreatic cancer)*. Many patients desired a personal patient-physician relationship: *“A bit of interest in the patient (…)*, *a few personal thoughts about life*, *whatever*, *that’s a good doctor*. *Whether that can be implemented in our system is a different matter*” *(P10b*, *male*, *aged 75 years*, *suffering from lung cancer)*. *“A good doctor is someone who makes sure he’s at the same level—if possible (laughs)” (P20a*, *female*, *aged 59 years*, *suffering from breast and ovarian cancer)*.

## Discussion

The present qualitative study revealed the patient’s view of palliative care. To our knowledge, this is the first study which sought to explore and identify patients’ previous knowledge of palliative care, their expectations and needs when being admitted to a palliative care unit, their experiences one week after admission and their perception of a good palliative care physician. This report is focused on a hitherto poorly researched topic, now addressed under difficult circumstances with patients who were severely ill [25]. However, our study participants had a median Karnofsky performance status scale (KPS) of 60%. In other words, they needed occasional assistance but were able to care for most of their personal needs [26]. Prior knowledge about hospices was common in our study group. The results of our study show that the patients could not distinguish hospices from palliative care units and lacked information about the purpose of a palliative care unit at the time of their admission. In Austria, palliative care units provide treatment ahead of discharge while hospices attend to patients in the final phase of their lives until death [27]. Some interviewees perceived the palliative care unit as a hospice or a unit where they would probably die, a few thought they were being admitted to the wrong unit, while some thought it was a unit for recuperation and symptomatic relief. The fact that 50% (10 out of 20) of patients were not informed about the exact purpose of their stay at the palliative care unit suggests that communication should be optimized and more education and information provided [17]. Many patients were feeling that a palliative care unit would be kind of a “last unit” or a “death ward”. Therefore, some patients might feel lost and hopeless when being admitted to a palliative care unit. Also, some physicians are concerned about decrements in the emotional well-being of their patients by admitting them to a palliative care unit. Since hospice beds in Austria are not regularly state funded, the large part of palliative care is undertaken in dedicated units integrated into comprehensive cancer centers. It is known that early palliative care should be encouraged and may even improve the prognosis [2, 5, 28]. Therefore, the patients should be given timely information about palliative care in order to improve their access to palliative care services and be admitted early to palliative care [29, 30]. The core competences in palliative care include communication, shared decision-making, symptom management, and preserving quality of life [31–34]. Another aim of this study was to explore the patient’s care needs from their point of view. Besides symptom management and honest and attentive communication, patients expressed a major supportive care need regarding help in social concerns. It was especially important to the patients to regulate financial issues, gain support for their home environment and unburden their caregivers. This highlights the importance for a deeper integration of social workers in palliative care units. Patient satisfaction and developing a patient-physician relationship also relates to available time resources [35]. Our study yielded findings concerning major social and emotional skills that patients expected of a palliative care physician: honesty, the ability to listen, taking time, being experienced in their field, speaking the patient’s language, being human, and being gentle. In addition to professionalism, the patients obviously desired a more personal relationship with staff. In our study, patients expressed their need for honest communication, which is in accordance to previous findings from the literature [36]. However, herein lies a barrier in the patient-physician communication, since some physicians still fear that by breaking bad news the patients will be discouraged and lose hope. Our recommendation is to adapt the quantity of information to the patient’s and caregivers individual desire [37]. The published literature about patient perspectives on prognosis and end-of-life issues emphasizes that some patients desire information while others do not. However, it is also known that the patient-physician relationship is not adversely affected by raising these issues [38–44]. The challenge is to be true and give hope without raising false hopes. Knowing about what patients do expect from a good palliative care physician as shown by our findings is very important. It should further encourage healthcare professionals in helping to alleviate patient’s fears by taking time, listening, communicating openly and in this way make it possible for the patients to redefine their hope on a goal other than cure [45]. Being compassionate, empathetic and attentive while interacting with patients and their caregivers should be regarded as a clinical skill and may be interpreted as a medical attitude. The second interview conducted after one week revealed that the patients perceived their stay in a palliative care unit as a largely positive experience. Due to this finding, healthcare professionals should inform their patients about the purpose of a palliative care unit in a timely manner and encourage them by telling them that many patients do benefit from a palliative care treatment. The study participants reported that their condition had improved because they received symptom management and were able to talk to members of the palliative care team. They also realized that the purpose of their admission was to achieve their discharge from the hospital. The fact that some patients were overwhelmed by too intensive treatment should raise the awareness of treating physicians to repeatedly reconsider their therapeutic strategies. Our findings provide solid evidence what patients do expect from a palliative care unit and that they mostly benefit from their admittance to the palliative care unit while their knowledge about the aim of palliative care units is still limited.

### Strengths and limitations of the study

The limitations of the present study include the fact that all of the patients were recruited from the single unit of a university-based hospital. Those from other palliative care units or patients treated by mobile palliative care teams were not included. Patients admitted to a hospital are in a dependent situation and may have withheld their criticism. Our study population consisted of German-speaking Austrian patients. Our data do not provide information about the perceptions and needs of migrant patients with a different cultural background. Besides, younger patients might have different needs. Patients in a dying process or patients with a mental impairment might have different requirements that they could not express. This study only addressed patients that were admitted to the palliative care unit and includes no data about what patients without this access would have needed. The strength of this study lies in the fact that, to minimize interviewer bias, all patients were interviewed by a medical student (A.K.) who was not identified as a team member. Given the fact that palliative care units are small units and patients are severely ill, 32 interviews were a satisfactory number. The transferability of data in qualitative research is limited. Since the present study was focused on cancer patients, the results cannot be generalized. Studies addressing the preferences of those with non-malignant disease are scarce [46]. Hence, the findings of this study may be used to underline the importance of addressing the needs of patients suffering from severe non-malignant diseases in future studies. The expansion of palliative care into other fields will become a matter of course. Involving palliative care patients in research is very important but also challenging [25]. In conformity with previous publications our study confirmed the patients’ willingness to participate in a qualitative study [47].

### Clinical Recommendations

The end of life is an inevitable phase in all our lives. The patients should be educated about palliative care before being admitted to a palliative care unit. Education materials such as brochures, booklets or videos should help the patients to prepare for their hospitalization. Leading clinical personnel should acknowledge the fact that taking time for the patient is a crucial clinical skill that should be encouraged. Therefore, the healthcare system should continue to expand the implementation of palliative care, uncover misconceptions, and reduce fear by appropriate information about the purpose and content of palliative care [48]. Furthermore, social workers are very important members of palliative care teams. Social workers may help the patients and their families to resolve difficulties and maintain an active social life [49, 50].

### Recommendations for Future Research

Further research on patients’ expectations is needed because palliative care is not only about end-of-life issues. Rather, it is about attention to detail. This topic warrants more attention not only to alleviate distress in patients receiving palliative care, but also to improve palliative care in clinical practice. In addition, patient’s perspectives might help palliative care teams to further improve the quality of their care and provide comfort to patients in the last phase of their lives. This would reduce barriers and allow further implementation of palliative care in general health care.

## Conclusions

This study identified patient’s perspectives on palliative care as well as qualities required of palliative care physicians as reported by the patients. The findings of the present investigation should help to educate future palliative care professionals. Healthcare professionals should provide information about palliative care and ask for the patient’s pre-existing knowledge about palliative care. A sensitive approach that takes the needs of patients into account will help to eliminate misconceptions about palliative care.

## Supporting Information

S1 Appendix Original German Quotations in Order of Appearance. (PDF)
