# Supplementary material for: What Makes a Good Palliative Care Physician? A Qualitative Study about the Patient’s Expectations and Needs when Being Admitted to a Palliative Care Unit
Source: PLoS One. 2016 Jul 7;11(7):e0158830. doi: 10.1371/journal.pone.0158830 (PMC4936709; doi:10.1371/journal.pone.0158830)
Supplement: S1 Appendix — (PDF) [file pone.0158830.s001.pdf]

Original German Quotations in Order of Appearance

Theme 1: Information about Palliative Care

Man liest oder hört etwas über über Hospize in der Zeitung oder im Fernsehen. Ich dachte eine Palliativstation, das ist das Gleiche.
(P01a, weiblich, 58 Jahre, Brustkrebs)

Ich habe nur mitbekommen, dass man an die Krankheitssituation anders herangeht und sich intensiver damit beschäftigt.
(P03a, männlich, 64 Jahre, Gehirntumor)

Ganz begriffen habe ich es nie. Vom Land, wo ich herkomme, ist „palliativ“ etwas, dass man mit dem Ende vom Ganzen verbindet. Aber anscheinend wollen die Menschen dir da helfen.
(P04a, weiblich, 53 Jahre, Brustkrebs)

Palliativ das ist eben, im Sprachgebrauch hat man so gesagt, „Palliativ - letztes Kammerl deines Lebens“. So ist es aber vielleicht gar nicht.
(P19a, weiblich, 79 Jahre, Pankreaskarzinom)

Ich habe überhaupt keine Ahnung gehabt. Ich habe auch jetzt noch keine Ahnung, was ich da mache und wie das da weiter geht.
(P02a, männlich, 73 Jahre, Lungenkarzinom)

Mir wurde nicht erklärt, was das ist. Da muss man fragen! (lacht)
(P09a, weiblich, 49 Jahre, Brustkrebs)

Theme 2: Supportive Care Needs

Dem sozialen Druck wird zu wenig Aufmerksamkeit geschenkt, denn diese Faktoren sind eine schreckliche Bürde für die Menschen.
(P03b, männlich, 64 Jahre, Gehirntumor)

Naja, ich habe gehört, das ist eigentlich eine Sterbestation. Manche überleben es und gehen wieder nach Hause und haben eine bessere Lebensqualität. Das wäre ja sehr erfreulich, ich hoffe, das gelingt mir auch.
(P07b, weiblich, 66 Jahre, Lungenkrebs)

Dass man mich ernst nimmt. Und zuhört ja. Das habe ich mir immer gewünscht. Und es war schon ein paar Mal so, dass ich gedacht habe, die Ärzte hören überhaupt nicht zu.
(P05a, weiblich, 42 Jahre, Dünndarmkarzinom)

Ein bisschen Zeit nehmen, zuhören, zuhören und ein bisschen Einfühlungsvermögen, aber nicht sagen, „Naja lange machen Sie es eh nicht mehr“.
(P19b, weiblich, 79 Jahre, Pankreaskarzinom)

Ja diese besonderen Eigenschaften sind so diese Kompetenz, das ist für mich sehr wichtig, auch das mitteilen, was passiert. Und was die Prognose ist, wenn ich schon frage, dann sollte ich es wissen. Und Ehrlichkeit und immer das Bemühen, doch das Beste für mich zu versuchen.
(P20a, weiblich, 59 Jahre, Brustkrebs, Ovarialkarzinom).

Im ersten Moment assoziiere ich mit Palliativmedizin den allerletzten Lebensabschnitt und das Faktum, dass man mir das Leben irgendwann einmal, wenn ich am Ziel bin, schmerzfrei gestalten kann.
(P10a, männlich, 75 Jahre, Lungenkrebs)

Theme 3: Being Treated on a Palliative Care Unit

Also jetzt verstehe ich unter einer Palliativstation, dass man fit gemacht werden sollte, dass man nach Hause kann. Ja, und dass man aber auch jederzeit wieder kommen kann, wenn es einem schlechter geht.
(P01b, weiblich, 58 Jahre, Brustkrebs)

Wieder kräftiger zu werden und an Hoffnung gewinnen - das wurde hier erreicht.
(P19b, weiblich, 79 Jahre, Pankreaskarzinom)

Ich denke man kümmert sich hier mehr um die Leute. Sie konzentrieren sich nicht nur auf deine Gesundheit alleine, sondern schauen, dass du hier zur Ruhe kommst, oder zumindest eine Pause hast. Sie unterstützen dich in Situationen wenn etwas gebraucht wird, zum Beispiel Psychologen, Diätberatung, oder was zu Hause nötig ist.
(P03b, männlich, 64 Jahre, Gehirntumor)

Ich habe es mir ärger vorgestellt. Ich habe geglaubt, dass da ärger Kranke sind, aber ich habe es mir ärger vorgestellt.
(P09b, weiblich, 49 Jahre, Brustkrebs)

Also ich habe mir so eine Station überhaupt nie vorstellen können und ich bin wirklich positiv überrascht, wie viel Mühe man sich da nimmt und wie bemüht die Leute da sind und wie da gearbeitet wird. Es ist eine andere Art als woanders und so sollte es wahrscheinlich sein. Ich denke, es ist richtig so.
(P07b, weiblich, 66 Jahre, Lungenkarzinom)

Dass man einmal sagt, wenn ich - wie oft bin ich auf der Onkologie - alle drei Wochen. Da wäre es gut gewesen wenn man gesagt hätte, „Hören Sie, wenn Sie sich schlechter fühlen, da gibt es da eine spezielle Station“, und so. Aber es war nie eine Rede davon. Sie haben kein Wort darüber verloren.
(P01b, weiblich, 58 Jahre, Brustkrebs)

Der Psychologe kommt, dann kommen die Ehrenamtlichen. Manchmal ist es schon fast zu viel.
(P13b, weiblich, 51 Jahre, Sarkom)

Nicht nur, „Ja ja, wir machen das“ und dann folgen keine Taten. Es wird etwas getan und das hilft, ja.
(P03b, männlich, Gehirntumor)

Wenn man sagt „Ich möchte mit einem Arzt sprechen“, dann ist da die ganze Woche jemand verfügbar der dir alles erklärt. (…) Einfach Zeit. Sie nehmen sich Zeit und erklären dir alles ausführlich.
(P05b, weiblich, 42 Jahre, Dünndarmkarzinom)

Theme 4: Qualities Required from Palliative Care Physicians

Ehrlichkeit. Da brauche ich nicht viel nachdenken. Das ist es, einfach Ehrlichkeit.
(P18a, männlich, 51 Jahre, Hautkrebs)

Gute Nachrichten vielleicht und ja, die Ärzte sind auch hier glaube ich ein bisschen so. Sie lachen viel (…), ja, die Ärzte sollen mehr lachen.
(P05b, weiblich, 42 Jahre, Dünndarmkarzinom)

Zuhören, kommunizieren, zuhören und menschlich sein. Zuhören ist ganz, ganz wichtig.
(P07b, weiblich, 66 Jahre, Lungenkarzinom)

Ein bisschen Zeit für Patienten. Ein bisschen mehr Zeit. Das gibt es nirgends. Man sieht, wenn man in den Ambulanzen ist, das ist alles überfüllt, wo können die sich Zeit nehmen. (…) Ich denke das ist von der Politik gesteuert. Die Arbeitsstunden werden immer gestrichen und es kommt nichts Vernünftiges dabei heraus. Ich verstehe diese Politik nicht.
(P08, weiblich, 68 Jahre, Pankreaskarzinom)

Ein gewisses Interesse am Patienten (…), ein paar persönliche Gedanken zum Leben, was auch immer, das ist ein guter Arzt. Ob das dann funktioniert in unserem System, das ist eine andere Sache.
(P10b, männlich, 75 Jahre, Lungenkarzinom)

Ein guter Arzt ist jemand, der sich auf dieselbe Stufe begibt, wenn möglich (lacht).
(P20a, weiblich, 59 Jahre, Brustkrebs, Ovarialkarzinom)
